# Immunogenicity Characterization of COVID-19 Vaccines: A Systematic Review and Meta-analysis

**DOI:** 10.1590/0037-8682-0661-2022

**Published:** 2023-01-23

**Authors:** Zanair Soares Vasconcelos, Hany Abdulateif Salem, Sâmia Pimenta Veiga, Fabiola Estefany Botelho de Lima, César Rogério da Silva Gonçalves, Eliane Carvalho dos Santos, Alba Regina Jorge Brandão, Kátia Nascimento Couceiro, Jorge Augusto de Oliveira Guerra, Maria das Graças Vale Barbosa Guerra

**Affiliations:** 1 Universidade do Estado do Amazonas, Programa de Pós-Graduação Stricto Sensu em Medicina Tropical, Manaus, AM, Brasil.; 2 Centro de Instrução de Guerra na Selva, Manaus, AM, Brasil.; 3 Egyptian Ministry of Health and Population, Cairo, Egypt.; 4 Faculdade Martha Falcão, Manaus, AM, Brasil.; 5 Laboratório Distrital Sul, Secretaria Municipal de Saúde de Manaus, Manaus, AM, Brasil.; 6 Universidade do Estado do Amazonas, Manaus, AM, Brasil.; 7 Fundação de Medicina Tropical “Heitor Vieira Dourado”, Manaus, AM, Brasil.

**Keywords:** COVID-19, SARS-CoV-2, Vaccines, Immunogenicity, Coronavirus vaccine

## Abstract

**Introduction:**

Immunogenicity has emerged as a challenge in the development of vaccines against coronavirus disease of 2019 (COVID-19). Immunogenicity is a determinant of the efficacy and safety of vaccines. This systematic review and associated meta-analysis summarized and characterized the immunogenicity of COVID-19 vaccines in randomized controlled trials (RCTs).

**Methods:**

Relevant RCTs were systematically sourced from different medical databases in August 2021. The risk ratios and mean differences with 95% confidence intervals were calculated.

**Results:**

Of 2,310 papers, 16 RCTs were eligible for review. These RCTs involved a total of 26,698 participants (15,292 males and 11,231 females). The pooled results showed a significant difference in the geometric mean titer between the vaccinated and control groups in favor of the vaccine group after 1 and 2 months of follow-up, for the young age group (18 - < 55y), and with different doses (P < 0.001). The difference in the older age group (>55y) was insignificant (P = 0.24). The seroconversion rate of spike neutralizing antibodies favored the vaccine groups 1 or 2 months after vaccination (P < 0.001). The seroconversion rate of the vaccine group was significantly different (P < 0.001) from that of the control group.

**Conclusions:**

Vaccination elicits immunogenicity in the follow-up period for all age groups and at low and large doses. Therefore, people should be encouraged to receive vaccines currently being offered. A boost dose has been asserted for the elderly.

## INTRODUCTION

Immunogenicity has emerged as a challenge in the development of vaccines against coronavirus disease 19 (COVID-19)^1^ ^,^ ^2^. Immunogenicity is a determining factor in the efficacy and safety of COVID-19 vaccines^3^ ^,^ ^4^ ^,^ ^5^ for combating severe acute respiratory syndrome coronavirus 2 (SARS-CoV-2). Over 200 COVID-19 vaccines were initially tested against SARS-CoV-2, of which only a few completed phase I and II clinical trials^6^. Immunogenicity is the ability of a foreign, non-self-substance, for example, an antigen, to activate the organism's immune system and provoke an immune response. The main mechanism of action of vaccination is to provoke an immune response (immunogenicity) against a specific antigen, for example, a virus or substance, to protect the organism from future harm when re-exposed to the same antigen^7^. Candidate vaccines can be grouped according to three major strategies: (1) nucleic acid-based vaccines, either ribonucleic acid (RNA-) or deoxynucleic acid (DNA-) based vaccines; (2) whole virus vaccines, either inactivated or live vaccines; and (3) subunit vaccines^8^. The first strategy involves the use of nucleic acid mRNA-based vaccines. It is a novel technology that utilizes a single-stranded RNA molecule carrying the coding sequences of the COVID-19 spike protein (S-protein) encapsulated in a lipid nanoparticle^9^ ^,^ ^10^. Another nucleic acid-based approach involves viral vector vaccines that exploit recombinant DNA techniques to clone the genes that encode the viral antigen S-protein. The second strategy includes whole-pathogen inactivated virus vaccines containing killed or inactivated whole vial particles or fragments^11^ ^,^ ^12^. That appeal to researchers because of the long-term success of polio vaccination. The third strategy, subunit vaccines, does not contain viable viral pathogen particles or any genetic material, thus enhancing safety. The subunit approach uses nanoparticles coated with the synthetic COVID-19 signature S-protein and an added adjuvant^13^ ^,^ ^14^. 

Although these vaccine technologies can elicit immunogenicity with presumed protectiveness, to the best of our knowledge, no comprehensive comparative study has addressed their immunogenicity through a meta-analysis. The vaccine that produces the highest immunogenicity has not yet been addressed. Therefore, we conducted a systematic review and meta-analysis study of published studies on available randomized controlled clinical trials (RCTs). The current study aimed to summarize and characterize the immunogenicity of the current COVID-19 vaccines. Hopefully, the results of this study could be a foundation for future studies to elicit more information regarding the immunogenicity of COVID-19 vaccines. 

## METHODS

The Preferred Reporting Items for Systematic Reviews and Meta-Analyses (PRISMA) statement guidelines^15^ were followed throughout the processing stages of this study. The processing stages were performed in accordance with the Cochrane Handbook for Systematic Reviews of Interventions^16^.

### Eligibility Criteria

Studies were included according to the following criteria: (1) studies with multiple age groups including (2) double-arm designs, (3) studies that included randomized controlled trials (RCTs), (4) studies published in English, and (5) the geometric mean titer (GMT) and seroconversion rate of spike neutralizing antibodies were selected as the outcome of the study to express the immunogenicity of the vaccine under investigation. Other immunogenicity data were excluded, including those on cellular immunity. Moreover, we excluded conference abstracts, unpublished data, studies written in a language other than English, *in vitro* studies, and duplicated papers, whether they included longer follow-up studies. 

### Outcomes

The outcomes are attributed to the spike neutralizing antibodies, including spike neutralizing antibody GMT after 1 and 2 months of follow-up, SARS-CoV-2 spike neutralizing antibody GMT for different age groups, SARS-CoV-2 spike neutralizing antibody GMT at different doses (low dose and high dose), seroconversion of spike neutralizing antibodies after 1 month of follow-up, seroconversion of spike neutralizing antibodies after 2-month of follow-up, and spike neutralizing antibody seroconversion rate.

### Endpoints

The endpoints included the GMT of spike neutralizing antibody after 1 month and 2 months of follow-up, the GMT of spike neutralizing antibodies according to age group (18 - <55 and > 55 years old) and dose (either low or high dose), and finally the seroconversion rate after one month and two months of follow-up. 

### Search Strategy and Study Selection

Using relevant keywords, PubMed, Scopus, Cochrane, Web of Science, Embase, and Science Direct databases were searched in August 2021. The search terms used included COVID-19, SARS-CoV-2, vaccines, and viral vaccines (Supplementary Material). 

The search results were independently screened by two authors: SPV and HAS. The study selection was based on the eligibility criteria. Exclusion of studies relied first on the paper’s title and then on the abstract, followed by full-text screening. The bibliographic references of the included studies were also manually screened to identify any other eligible studies that may have been missed in the previous stages. Data were extracted and collected on an electronic spreadsheet for further processing. The extracted data included demographic information, age groups, sex, dose groups, and the GMT and seroconversion rate of the spike neutralizing antibodies in the vaccinated and control groups. A third author (ZSV) resolved any disagreements between the other two authors. 

### Quality Assessment

The risk of bias was evaluated using the Cochrane Handbook for Systematic Reviews of Interventions, version 5.1.0^17^. The six domains of the Cochrane risk-of-bias tool included selection bias, performance bias, detection bias, attrition bias, reporting bias, and other biases. One or more items were assessed within each domain, which could cover different aspects of the domain of concern. Performance bias was addressed by blinding participants and personnel. Detection bias was addressed by blinding outcome assessors. Each bias domain was recorded as low-risk, high-risk, or unclear risk). Further details are included in the protocol (Supplementary material).

### Data Extraction

Data were obtained from text, tables, and supplementary data. We focused on the outcome measures that included spike neutralizing antibody GMT after 1 month and 2 months of follow-up, spike neutralizing antibodies according to age group (18 - <55 and > 55 years), spike neutralizing antibodies according to dose (either low or high dose), and finally the seroconversion rate after 1 month and 2 months of follow-up. A summary of the baseline patient characteristics is presented in Supplementary Table 1.

### Statistical Analysis

This meta-analysis was conducted using the Review Manager computer program (RevMan, version 5.4 Copenhagen: The Nordic Cochrane Centre, The Cochrane Collaboration, 2014). Regarding the study outcomes, a risk ratio (RR) with a 95% confidence interval (CI) was used for dichotomous variables, whereas the mean difference (MD) and 95% CI were presented for continuous variables. The 95% confidence interval (CI) between the intervention and control groups was recommended by Cochrane. Cochrane’s *P*values and I^2^were tested to examine the heterogeneity among the studies^16^. High heterogeneity was most likely due to clinical and methodological factors, so the random effect model was adopted in this meta-analysis even if I^2^was small. Funnel plots and the Egger regression test could not be performed because of the limited number of included trials^18^. In addition, sensitivity analysis was performed by sequentially deleting trials to check the stability of the outcomes. No significant effect was observed after performing the sensitivity analysis because of the high variation between the included studies. The heterogeneity remained high (I2 > 50%) after sensitivity analysis. Further details are included in the protocol (Supplementary material).

## RESULTS

### Literature Search Results

**FIGURE 1:**
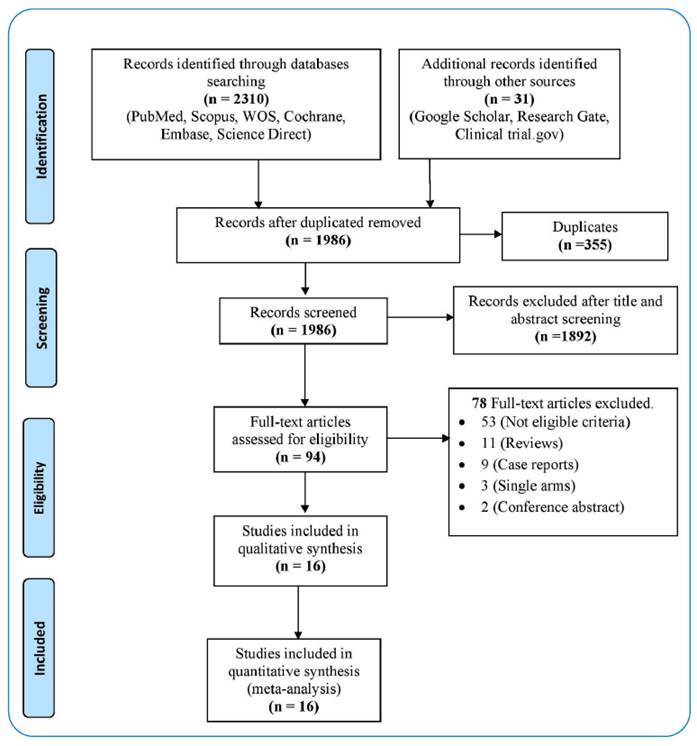
PRISMA flow diagram of the included studies.

The initial search resulted in 2,310 papers from six databases: PubMed, Cochrane, Scopus, Web of Science (WOS), Embase, and Science Direct. Of these 2,310 papers, 355 were excluded because of duplication. Subsequently, 1,986 papers underwent title and abstract screening and 1,892 were excluded because they did not meet the inclusion criteria. The remaining 94 papers were subjected to full-text screening. A total of 16 studies were finally included for the final qualitative and quantitative analysis: three papers on nucleoside-modified mRNA vaccines^9^ ^,^ ^19^ ^,^ ^20^, five papers on DNA-based vaccines^5^ ^,^ ^21^ ^,^ ^22^ ^,^ ^23^ ^,^ ^24^, three papers on subunit vaccines^25^ ^,^ ^26^ ^,^ ^27^, and five papers on whole pathogen inactivated virus vaccines^4^ ^,^ ^23^ ^,^ ^28^ ^,^ ^29^ ^,^ ^30^. Exclusion from the full-text screening was based on the following reasons: fifty-two papers with non-eligibility criteria, 11 papers were reviews (non-comparative studies not eligible for extraction), nine papers were case reports, three papers followed the single-arm design, and the final two papers were conference abstracts (Figure 1). 

### Risk of Bias Assessment of the Included Studies

**Supplementary Figure 1:**
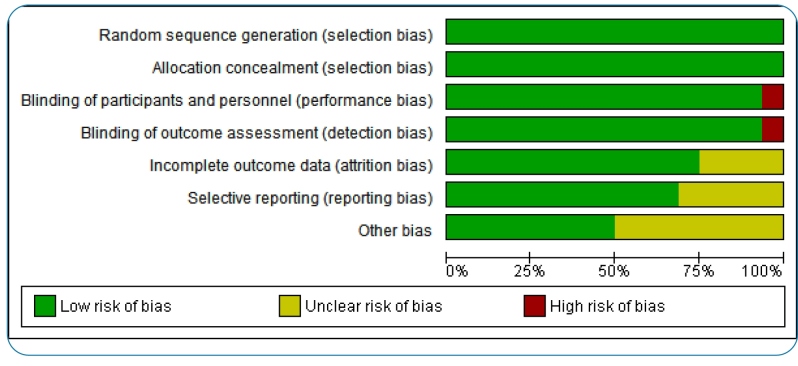
Cochrane risk of bias assessment graph of the included trials.

**Supplementary Figure 2:**
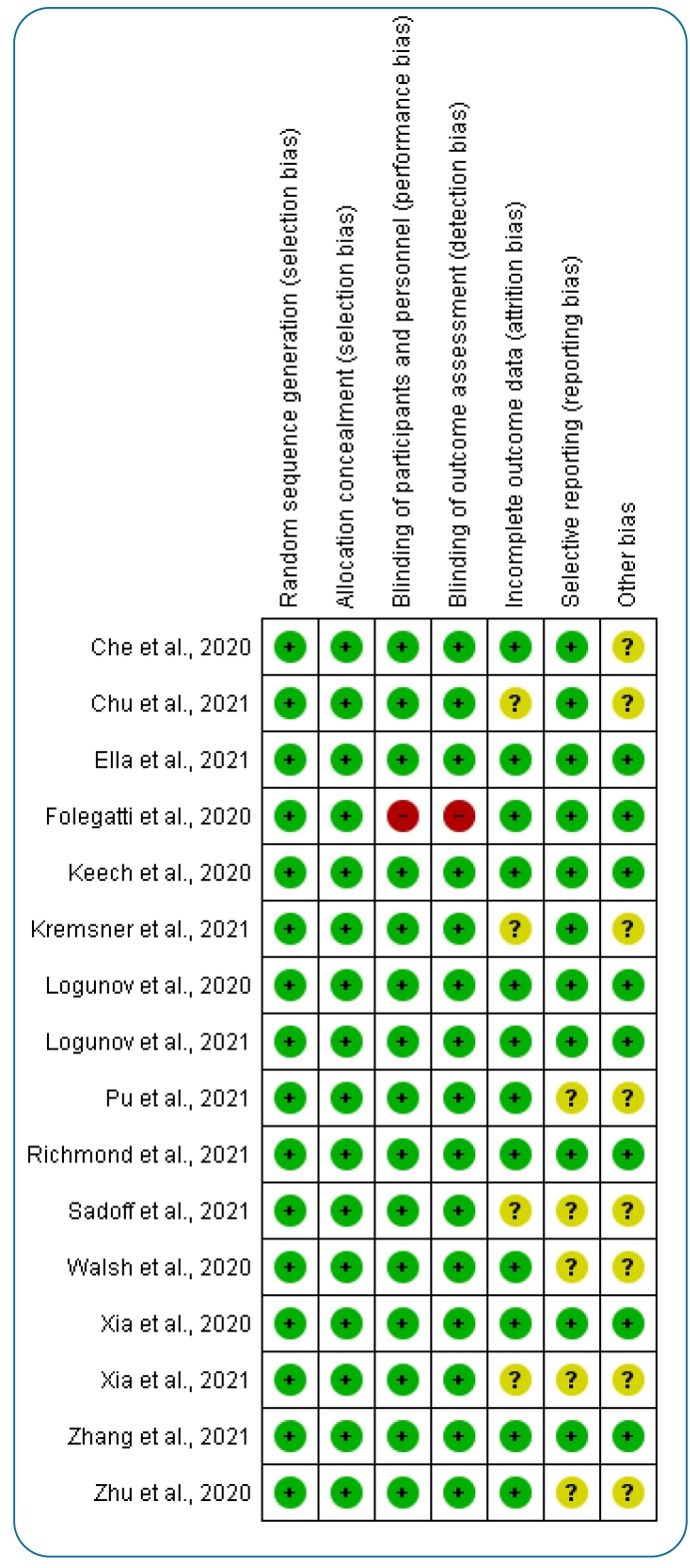
Cochrane risk of bias assessment graph of the individual trials.

All eligible studies reported allocation concealment and random sequence generation with no selection bias. Folegatti et al. is the only study that has a high risk of performance bias (blinding of participants and personnel) and detection bias (blinding of outcome assessment)^21^. Four studies showed an uncertain attrition bias^9^ ^,^ ^19^ ^,^ ^22^ ^,^ ^23^. Five studies showed selective reporting bias^20^ ^,^ ^22^ ^,^ ^23^ ^,^ ^24^ ^,^ ^29^. Other biases were unclear in 8 studies^9^ ^,^ ^19^ ^,^ ^20^ ^,^ ^22^ ^,^ ^23^ ^,^ ^24^ ^,^ ^28^ ^,^ ^29^. Therefore, the overall quality of the included studies was considered high, and there was a low risk of bias (Supplementary Figure 1,Supplementary Figure 2).

### Descriptive Data

The total number of participants was 26,698 (15,292 males, 11,231 females). Eligible studies were conducted in nine countries, including the USA, the UK, China, Australia, Russia, Belgium, Germany, South Africa, and India (Supplementary Table 1). It was found that 3 papers were investigating nucleoside-modified mRNA-based vaccines, 5 papers studying DNA sequences-based vaccines, 5 papers investigating whole pathogen vaccines, and 3 papers investigating subunit-based vaccines (Supplementary Table 1). The numbers of participants in the vaccine and control groups are summarized in Table 1 and correspond to each outcome analysis. Some studies were found to have more than one dataset according to the age of the participants, dose schedules, and dose regimens. The datasets are summarized in Supplementary Table 2.

**TABLE 1:** Summarization of analysis outcomes.

Outcomes	Subgrouping	MD/ RR	CI		P-Value	Number of each group	
			Lower limit	Upper limit		vaccine	Control
**Spike neutralizing antibody (nAB) GMT**	1 month follow-up	219.19	106.78	331.6	**0.0001**	17,241	5,956
	2 months follow-up	1600.03	1537.08	1662.99	**< 0.00001**	302	171
	Total	489.81	375.49	604.13	**< 0.00001**	17,543	6,127
**Spike neutralizing AB according to age group**	18 - <55 yr age group	350.35	233.63	467.07	**< 0.00001**	16,967	5,753
	> 55 yr age group	859	-581.77	2299.77	0.24	305	175
	Total	489.08	366.44	611.72	**< 0.00001**	17,272	5,928
**Spike neutralizing AB according to dose**	Low dose (age 18 - <55 yr)	57.44	46.84	68.04	**< 0.00001**	16,695	5,671
	High dose (Age 18 - <55 yr)	452.04	403.92	500.17	< 0.00001	203	195
	Low dose (age > 55 yr)	240.08	204.12	276.04	**< 0.00001**	149	175
	High dose (age > 55 yr)	230.38	192.08	268.69	**< 0.00001**	156	175
	Total	97.25	87.62	106.88	**< 0.00001**	17,203	6,216
Spike neutralizing antibodies seroconversion	Total	95.20	64.64	125.77	**< 0.00001**	665	406

**MD:** Mean difference, **RR:** Risk Ratio, **CI:** Confidence interval, **AB:** Antibody.

### Outcomes

### Spike neutralizing antibody GMT after 1 & 2 months of follow-up

**FIGURE 2:**
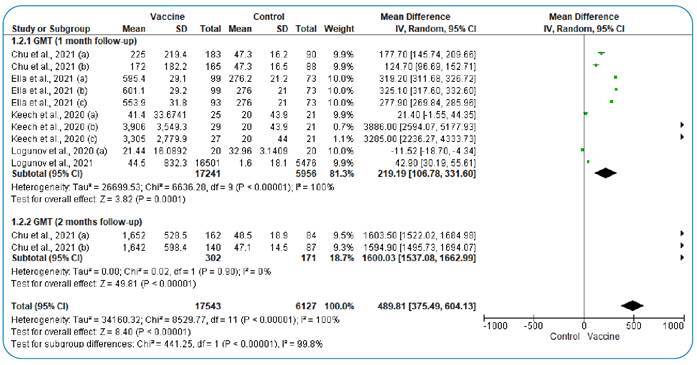
Forest plot of mean difference in SARS-CoV-2 spike neutralizing antibody genomic mean titer after 1 & 2 months of follow-up.

After one month of follow-up, the pooled analysis of the included studies showed a significant difference between the vaccinated group and the control group regarding GMT after vaccination (MD = 219.19; 95% CI: 106.78, 331.6, P <0.001). After two months of follow-up, the pooled results showed a significant difference between the vaccinated and control groups (MD = 1,600.03; 95% CI:1,537.08, 1,662.99, P < 0.001). The pooled studies were heterogeneous (I^2^ = 100%, P < 0.001), and the heterogeneity could not be resolved owing to the high variation between the groups (Figure 2).

### SARS-CoV-2 spike neutralizing antibody GMT for different age groups

**Supplementary Figure 3:**
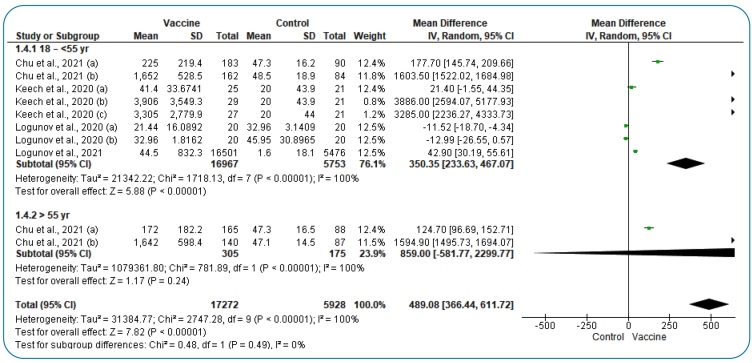
Forest plot of mean difference in the spike neutralizing antibodies according to age group.

The pooled analysis of the included studies showed a significant difference between the vaccinated and control groups for younger and older age groups (MD = 489.08, 95% CI:366.44, 611.72, P *<* 0.001*).* For younger age groups (18-55 years), there was a significant difference between the vaccinated group and the control group (MD = 350.35; 95%CI: 233.63, 467.07, P < 0.001); however, no significant difference was detected in the older age group (> 55 years) between the vaccinated group and the control group (MD = 859.00; 95% CI: -581.77, 2,299.77, P = 0.24). The pooled studies were heterogeneous *(*I^2^ = 100% P *<* 0.001)*,* and the heterogeneity could not be resolved owing to the high variation between the groups (Supplementary Figure 3)

### SARS-CoV-2 spike neutralizing antibody GMT in different doses (low dose and high dose)

**Supplementary Figure 4:**
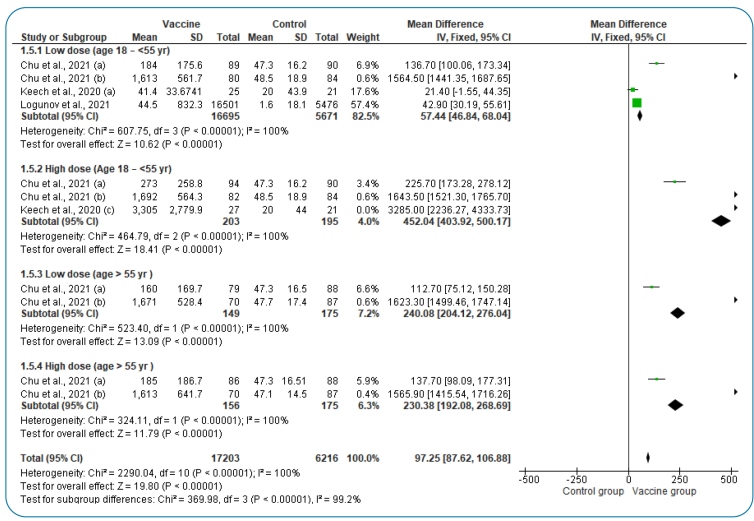
Forest plot of mean difference in the spike neutralizing antibodies according to high or low dose in different age groups.

Regarding the low vaccine dose in the younger age group, the analysis showed significant results (MD = 57.44; 95% CI:46.84, 68.04, P < 0.001). In addition, regarding the low dose for the older age group, the analysis showed significant results (MD = 452.04, 95% CI:403.92, 500.17; P < 0.001). For higher doses in both age groups, the results were significant between the vaccinated and control groups (MD = 240.08, 95% CI:204.12, 276.04, P < 0.001) (MD = 230.38, 95% CI 192.08, 268.69, P < 0.001). The overall pooled results of the included studies showed significant GMT results between the vaccinated participants and the control group (MD = 97.25; 95% CI: 87.62, 106.88, P < 0.001). The pooled studies were heterogeneous (I^2^ = 100%, P < 0.001), and the heterogeneity could not be resolved owing to the high variation between the groups*.* (Supplementary Figure 4)**.**

### Spike neutralizing antibodies seroconversion

**FIGURE 3:**
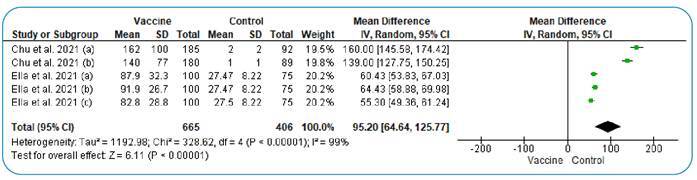
Forest plot of mean difference (MD) in the spike neutralizing antibodies seroconversion rate.

The pooled analysis of the included studies showed a significant difference between the vaccinated and control groups regarding seroconversion after one or two months of follow-up (MD = 95.20; 95% CI: [64.64, 125.77], P < 0.001), favoring the vaccinated group over the control group. The pooled studies were heterogeneous (I2 = 99%, P<0.001). Heterogeneity could not be resolved due to the high variation between the groups (Figure 3).

## DISCUSSION

The plethora of emerging vaccines against SARS-CoV-2 has raised the question of immunogenicity being a detrimental factor that confers protection against COVID-19^1^ ^,^ ^2^. The serum of patients with COVID-19 demonstrates high levels of neutralizing antibodies^2^ that have been correlated with the severity of COVID-19 infection^2^ and protection against SARS-CoV-2^31^ ^,^ ^32^. Moreover, the level of immunoglobulin G against the spike protein receptor-binding domain was found to correlate with spike neutralizing antibodies against SARS-CoV-2 in the sera of patients with COVID-19^33^. A high level of spike neutralizing antibodies was correlated with a reduction in viral load in patients with COVID-19^34^ ^,^ ^35^. Developed vaccines have been reported to retain the ability to elicit neutralizing antibodies against SARS-CoV-2, along with a high proportion of seroconversion^22^ ^,^ ^32^. Therefore, it was concluded that the neutralizing antibodies have implications for the development of a protectively effective vaccine against SARS-CoV-2^2^. To minimize the death toll of COVID-19, emergency use authorization was approved by the US Food and Drug Administration^36^ and the Department of Health and Human Services for some COVID-19 vaccines. However, the different aspects of vaccine efficacy, immunogenicity, cost, and untoward effects (early and remote) have not been comprehensively and thoroughly investigated^37^.

Therefore, a comprehensive evaluation of the datasets through a systematic review and meta-analysis would compensate for this gap in vaccine research. Given the need for a comprehensive, thorough, and punctual evaluation of the immunogenicity of different types of vaccines, the current study, a systematic review, and a meta-analysis focused on datasets of the spike protein and neutralizing antibodies as determinants of the immunogenicity (the outcomes) of the vaccines^38^. This expanded systematic review summarizes the available information on vaccine immunogenicity based on different parameters.

### Summary of the Results

The purpose of the current study is to summarize the available data on the immunogenicity of the current vaccines (Supplementary Table 1) in phase I/II, as reported in RCTs, as a detrimental factor of vaccine protection. The current study included 16 RCTs, which encompassed 26,698 participants. 

For immunogenicity, there was a significant increase in the spike protein-neutralizing antibody one and two months after vaccination, with a remarkable increase in the MD after the second month. Moreover, the seroconversion rate increased significantly as well. Therefore, a second booster dose was confirmed. 

While spike neutralizing antibodies showed a significant increase in the younger age group, spike neutralizing antibodies showed no significant increase in the older age group. However, irrespective of the vaccine dose, spike neutralizing antibodies significantly increased in both age groups. Moreover, 1 and 2 months of follow-up showed a significant spike in neutralizing antibody levels, with a rapid increase in the MD of spike neutralizing antibodies.

### Potential Bias

The included studies were of high quality, and the bias was low. Consequently, the derived results are believed to be reliable^17^.

### Context of this Review

Immunogenicity develops in the first month after vaccination, and this increase is remarkably augmented after the second month and the second (higher) dose. Immunogenicity was maintained for one and two months of follow-up, which suggests extended protection against COVID-19. The younger age group responded dramatically to the vaccination and showed a remarkable increase after receiving the second (higher) dose. Immunogenicity in older age groups requires further attention. These unfavorable results can be attributed to immunosenescence, which hinders the innate and adaptive immune response with an increase in age^39^. 

### Agreement and Disagreement with Literature

A systematic review conducted to evaluate the immunogenicity of SARS-CoV-1 and 2 and MERS-CoV concluded that inactivated and adenoviral-vector-based techniques were able to elicit robust antibody responses, especially in older and immunocompromised participants, while a single dose of mRNA1273 SARS-CoV-2 vaccine produced the highest antibody titer level^37^. Our study concurred with the findings of McDonald et al^37^ as the MD of spike neutralizing antibodies in the aged group was not statistically significant. The mRNA-based vaccine ( BNT162b2 vaccine by Pfizer-BioNTech and mRNA-1273 vaccine from Moderna) elicited a high immunogenic response in the elderly, followed by the ChAdOx1 nCoV-19 vaccine (AstraZeneca)^40^. In our analysis, although the MD of spike neutralizing antibodies was statistically significant, irrespective of age and dose, the antibody response in the elderly was not promising, even at high doses. It is worth noting that antibody responses in the elderly were less correlated with immune protection against viral influenza infection, and cell-mediated immune responses should be used instead^41^. 

It was concluded that the RNA-based vaccine showed a high level of neutralizing antibodies after one month of the first and second doses, and a high level was noticed with the adenovirus-vectored COVID-19 vaccine (Johnson & Johnson) with only one dose^42^. Contrary to the literature, the subunit vaccine NVX-CoV2373^27^ showed the highest MD of spike neutralizing antibodies in the first month, and the mRNA-based vaccine mRNA-1273^19^ showed a considerably high difference in the second month. The ineligibility of the data for extraction may explain this contradiction. However, the mRNA-based vaccine, mRNA-1273, resulted in the highest level of neutralizing antibody seroconversion rate after one month (Table 1) or the second month. 

Compared with other types of vaccines^43^, DNA-based vaccines have enhanced immunogenicity with fewer side effects attributed to antibody reactions. Notwithstanding, the overall seroconversion rate showed a significant increase, especially with the whole pathogen inactivated vaccine BBV152^4^. It has been claimed that vaccines based on the whole virus or complete spike protein may cause a hyperimmune response or post-immunization enhanced infectivity^44^. Therefore, eliciting immune responses should be integrated into the clinical safety of the response^43^.

### Strengths and Limitations of the Review

To the best of our knowledge, the current study is the first systematic review and meta-analysis to summarize the characteristics of immunogenicity of current COVID-19 vaccines. The inclusion of RCT only enhances the strength of the study, as well as the high number of participants recruited from the eligible papers. 

One substantial limitation was the heterogeneity among the included studies. The difference in the type of vaccines and the wide variation in the characteristics of the populations, including ethnicity, can explain the high variability between the vaccinated and control groups, and thus, heterogeneity. However, applying the random effects model and dividing the outcomes into subgroup analyses was carried out to manage the challenge of heterogeneity. 

Moreover, the data presented in some studies were not eligible for extraction, which led to the contraction of the datasets. Furthermore, the wide diversity of participants in the included studies may have caused bias in the results. It should be noted that the attribution of neutralizing antibodies to antigen-specific T-cell responses and disease severity is not well understood. Therefore, the results of the present study should be considered within this limitation. 

## RECOMMENDATIONS

These three categories of vaccines successfully stimulated the human immune system against the COVID-19 antigen and elicited presumed protective immunity. Therefore, taking any of the vaccines in these categories will enhance protection against SARS-CoV-2 infection. Moreover, it is recommended that the younger age group, from 18 to 60 years, take the vaccine because of the high response of their immune system. The older age group, over 60 years, may show a slow response rate and low level of immunity; therefore, the second dose would boost immunity and increase the chance of protection. 

All included RCTs investigated the immune response within a short period (nearly 2 months). Evaluation of the long-term immune response to various types of vaccines (6 months to 1 year) is needed to detect the solidity of the vaccinations and help us make further plans. The heterogeneity among RCTs calls for standardized RCT studies regarding data collection and reporting. Finally, extended standardized research is needed to highlight the protective effects of the elicited immunogenicity of current vaccines against other SARS-CoV-2 variants. 

## CONCLUSION

It is worth noting that all the vaccines in the three categories can elicit human body immune responses. Regardless of the type of vaccine, the immune system is sensitized against SARS-CoV-2 infection. Therefore, current vaccines can be protective tools for controlling the COVID-19 pandemic and preventing further fatalities. As no vaccine was proven to be more immunogenic, and hence more protective, people should be encouraged to receive the available vaccine in their country. The older age group is worth monitoring for the response of their immune system. 

## SUPPLEMENTARY INFORMATION

### Registration

This review has not been registered yet. No amendments have been made so far. The protocol can be found under the “Submitting Information” section.

### Data and material availability

All data are available within the manuscript and supplement.

## SUPPLEMENTARY MATERIAL

Keywords and search strategy for identification of studies:

The search will be conducted by using the databases PubMed, Scopus, Web of Science, Cochrane, Embase, Science Direct, and Google Scholar using the following keywords: “COVID 19 vaccine” AND “Antibodies” OR “Immunoglobulins” (“COVID 19 Vaccines “OR “Vaccines, COVID-19” OR “COVID-19 Virus Vaccines “OR “COVID 19 Virus Vaccines” OR “Vaccines, COVID-19 Virus” OR “Virus Vaccines, COVID-19” OR “COVID-19 Virus Vaccine” OR “COVID 19 Virus Vaccine” OR “Vaccine, COVID-19 Virus” OR “Virus Vaccine, COVID-19” OR “COVID19 Virus Vaccines” OR “Vaccines, COVID19 Virus” OR “Virus Vaccines, COVID19” OR “COVID19 Virus Vaccine” OR “Vaccine, COVID19 Virus” OR “Virus Vaccine, COVID19” OR “COVID19 Vaccines” OR “Vaccines, COVID19” OR “COVID19 Vaccine” OR “Vaccine, COVID19” OR “SARS-CoV-2 Vaccines” OR “SARS CoV 2 Vaccines” OR “Vaccines, SARS-CoV-2” OR “SARS-CoV-2 Vaccine” OR “SARS CoV 2 Vaccine” OR “Vaccine, SARS-CoV-2” OR “SARS2 Vaccines” OR “Vaccines, SARS2” OR “SARS2 Vaccine” OR “Vaccine, SARS2”).

**Types of included studies:**

**1. Inclusion criteria:**

- Studies including patients undergoing COVID vaccination.
- Studies written in English.
- Studies published in 2020 and 2021.
- Studies reporting the genomic mean titer (GMT) of nAB and seroconversion rates.
- Prospective randomized controlled trials.
- Human studies.

**2. Exclusion criteria**

- Publications including conference abstracts, case reports, case series, review articles, and letters to the editor.
- Studies not written in English.
- Studies with incomplete outcome data.
- Studies with selective outcome reporting.

**Types of included participants:**

Patients undergoing COVID vaccination with no restriction for a specific vaccine.

**Statistical considerations:**

Search results will be uploaded to the systematic review manager software, manually screened for eligibility, and then included. A PRISMA flowchart was produced based on the search results and the inclusion/exclusion criteria^1^.

To facilitate the assessment of the possible risk of bias for each study, information was collected using the Cochrane Collaboration Tool for Assessing the Risk of Bias^2^.Heterogeneity will be explained and, when necessary, a sensitivity analysis will be performed based on methodological quality and random effect versus fixed effect modeling. After pooling the collected data from the included studies, the relative risk of each of the intended outcome measures of interest was calculated with the aim of reaching a satisfactory conclusion.

**Statistical Package:**

Data will be collected, summarized, and reported on data collection sheets. Data were entered into Microsoft Excel spreadsheets with appropriate tabulation and graphical presentation and analyzed using Review Manager software (RevMan) version 5.4.

**Statistical Analysis:**

This meta-analysis was conducted using the Review Manager computer program (RevMan, version 5.4 Copenhagen: The Nordic Cochrane Centre, The Cochrane Collaboration, 2014). Regarding the study outcomes, a risk ratio (RR) with a 95% confidence interval (CI) was used for dichotomous variables, whereas the mean difference (MD) and 95% CI were presented for continuous variables. 95% confidence intervals (CI) between the intervention and control groups, as recommended by Cochrane.

Cochrane’s P-values and I^2^ were tested to examine heterogeneity among the 16 studies^2^. High heterogeneity was most likely due to clinical and methodological factors, so the random effect model was adopted in this meta-analysis even if I^2^ was small. Funnel plots and the Egger regression test could not be performed because of the limited number of included trials18^3^. In addition, a sensitivity analysis was performed by sequentially deleting the trials to check the stability of the primary outcomes.

Continuous numerical data in the form of means and standard deviations were described as quantitative data. Qualitative variables are presented in the form of frequency and percentages. The significance level (P) indicated the significance of the calculated tests. When the P-value was greater than 0.05, the result was considered insignificant. The result was considered significant if P < 0.05, and highly significant when P <0.001. Statistical significance was defined as a two-tailed p-value of < 0.05.

In accordance with the conventional acceptance of statistical significance at a P-value of 0.05 (or 5%), the CI was frequently calculated at a confidence level of 95%. In general, if an observed result is statistically significant at a P-value of 0.05, then the null hypothesis should not fall within the 95% CI. Calculating P-values depended on the deviation between the observed value and a chosen reference value, given the probability distribution of the statistic, with a greater difference between the two values corresponding to a lower p-value. The P-value was directly linked to the test statistic z=b/sb, which follows a standard normal distribution with mean 0 and unit variance, where b is an estimator of β and sb is the estimated standard error of b for any study included in the meta-analysis.

Heterogeneity was assessed using the I^2^statistic, which states the percentage of variability in effect estimates due to heterogeneity rather than chance. Thresholds for the interpretation of heterogeneity were based on Cochrane guidelines as follows:0% = no heterogeneity; > 0-40%: might not be important; 30-60%, moderate heterogeneity; 50-90%, substantial heterogeneity; 75-100%, considerable heterogeneity.

**Risk of Bias Assessment of the Included Studies**

Based on the Cochrane risk of bias tool found in chapter 8.5 of the Cochrane Handbook for Systematic Reviews of Interventions 5.1.0, random sequence generation, allocation concealment, selective reporting, and attrition bias met the standard criteria and had a low risk of bias. We assessed publication bias by investigating funnel plot symmetry and performing Egger’s test using Review Manager software version 5.4. Two reviewers independently assessed the risk of bias using the Cochrane Collaboration tool for the risk of bias domains. Regarding funnel plot asymmetry, we used a linear regression approach to measure funnel plot asymmetry on the natural logarithm scale of the odds ratio. If there is asymmetry, with smaller studies showing effects that differ systematically from larger studies, the regression line will not run through origin. In contrast to the overall test of heterogeneity, the funnel plot asymmetry test assesses a specific type of heterogeneity and provides a more powerful test in this situation. However, any analysis of heterogeneity depends on the number of trials included in a meta-analysis, which is generally small, limiting the statistical power of the test.

## Appendix

**SUPPLEMENTARY TABLE 1:** Summary of the included studies.

Study ID	Study design	Trial phase	Type of vaccine	Generic name vaccine for SARS Cov2	Developer company	Place of the study
Che et al., 2020	RCT	II	whole inactivated vaccine	Number 2020L00020 &		**China**
Chu et al., 2021	RCT	II	Nucleoside-modified mRNA	mRNA-1273	Moderna	**USA**
Ella et al., 2021	RCT	I	Whole pathogen inactivated virus	BBV152	Bharat Biotech’s	**India**
Folegatti et al., 2020	RCT	I/II	DNA sequences integrated into a modified safe adenovirus	ChAdOx1 nCoV-19	Oxford-AstraZeneca	**UK**
Keech et al., 2020	RCT	I/II	Subunit vaccine (no viable particles)	NVX-CoV2373	Novavax	**Australia**
Kremsner et al., 2021	RCT	I	mRNA-lipid nanoparticle vaccine	CVnCoV vaccine	CureVac N.V., Tübingen, Germany	**Hannover, Munich and Tübingen, Germany, and Ghent, Belgium**
Logunov et al., 2020	RCT	I/II	Vector-based heterologous prime-boost vaccine	rAd26 and rAd5	Gamaleya	**Russia**
Logunov et al., 2021	RCT	III	DNA sequences integrated into a modified safe adenovirus	rAd26 & rAd5	Gamaleya	**Russia**
Pu et al., 2021	RCT	I	Whole pathogen inactivated virus	CTR20200943 & NCT04412538	IMB-CAMS	**China**
Richmond et al., 2021	RCT	I	Subunit vaccine	SCB-2019	Clover Biopharmaceuticals, Chengdu, China	**Australia**
Sadoff et al., 2021	RCT	I/II a	DNA sequences integrated into a modified safe adenovirus	Ad26	-	**Belgium and the USA**
Walsh et al., 2020	RCT	I	Nucleoside-modified mRNA	BNT162b2/1 mRNA	Pfizer	**USA**
Xia et al., 2020	RCT	I/II	DNA sequences integrated into a modified safe adenovirus	rAd5 -vectored vaccine	-	**China**
Xia et al., 2021	RCT	I/II	Whole pathogen inactivated virus	BBIBP-CorV	Sinopharm	**China**
Zhang et al., 2021	RCT	I/II	Whole pathogen inactivated virus	CoronaVac	Sinovac Life Sciences (Beijing, China)	**China**
Zhu et al., 2020	RCT	II	DNA sequences integrated into a modified safe adenovirus	rAd5 -vectored vaccine	University of Oxford (Oxford, UK)	**China**

## Appendix

**SUPPLEMENTARY TABLE 2:** Dataset summary for the included studies.

Study ID	Dataset	Vaccination schedule (days)	Dose	Age groups
**Walsh et al. 2020**	(A)	7 days	BNT162b1 (100 ug)	18-55 Years
	(B)	14-28 days	BNT162b1 (30 ug)	65-85 Years
**Chu et al. 2021**	(A)	14 days	Low dose mRNA-1273 (50 ug)	18 - <52 years
	(B)	28-29 days	High dose mRNA-1273 (100 ug)	> 55 years
	(C)	57 days	mRNA-1273 (50, 100 ug)	> 18 years
**Ella et al. 2021**	(A)	0-7 days	BBV152 3 µg	≥18 to ≤25
	(B)	14-21 days	BBV152 6 µg	≥26 to ≤40
	(C)	> 42 days	BBV152 6 µg with Algel	>40 to ≤55
**Keetch et al. 2020**	(A)	7 days	0	30.3±10.92
	(B)	7 -14 days	Low dose	29.5±7.99
	(C)	7-21 days	High dose	27.2±9.38
**Logunov et al. 2020**	(A)	Day 28 post first dose	ChAdOx1 (one dose)	18 to <65
	(B)	Day 28 post second dose	ChAdOx1 (Two doses)	18 to <65
